# Supplementary material for: Shuttling a single charge across a one-dimensional array of silicon quantum dots
Source: Nat Commun. 2019 Mar 5;10:1063. doi: 10.1038/s41467-019-08970-z (PMC6401174; doi:10.1038/s41467-019-08970-z)
Supplement: Supplementary file 1 — Supplementary Information [file 41467_2019_8970_MOESM1_ESM.pdf]

Supplementary Information:
Shuttling a single charge across a one-dimensional array of silicon quantum dots

A. R. Mills,[1] D. M. Zajac,[1] M. J. Gullans,[1] F. J. Schupp,[1] T. M. Hazard,[1] and J. R. Petta[1]

[1]*Department of Physics, Princeton University, Princeton, New Jersey, USA*

Supplementary Discussion

When the quantum dot array is tuned away from all interdot charge transitions, its ground state has an effective classical electrostatic description in which each quantum dot is replaced by a conductor that is constrained to carry integer charge.[1] In this formulation we can write the system Hamiltonian in terms of two capacitance matrices $\boldsymbol{C}$ and $\boldsymbol{C}_V$

$$H = \sum_{ij} v_{ij} n_i n_j + \sum_i (\mu_i - \mu_{0i}) n_i, \tag{1}$$

$$v_{ij} \equiv \frac{e^2}{2}[\boldsymbol{C}^{-1}]_{ij},\ \mu_i \equiv e\alpha_1 u_i,\ \mu_{0i} = e\alpha_1 \sum_j G_{ij} V_{\mathrm{P}j}^{\mathrm{off}} = e\alpha_1 u_i^{\mathrm{off}}, \tag{2}$$

$$u_i \equiv \sum_j G_{ij} V_{\mathrm{P}j},\ G_{ij} \equiv -\frac{1}{\alpha_1}\sum_k [\boldsymbol{C}^{-1}]_{ik} C_V^{kj}, \tag{3}$$

where n_i is the dimensionless total electron number operator on dot i and e is the negative electron charge. The v_{ij} term has units of energy and describes the Coulomb interaction between electrons on dots i and j. The dimensionless matrix G_{ij} is used to convert between plunger gate voltages $V_{\mathrm{P}i}$ and the virtual gate voltages u_i with the convention $G_{11} = 1$. The positive dimensionless lever arm for dot 1, $\alpha_1 \equiv -[\boldsymbol{C}^{-1}\boldsymbol{C}_V]_{11}$ is used to convert from the virtual gate voltages u_i to the chemical potential μ_i of dot i. The lever arm for dot i is given by $\alpha_i = \alpha_1 G_{ii}$. Diagonal elements of $\boldsymbol{C}$ represent the total capacitance of each dot and the off-diagonal terms are the negative of the cross-capacitances between dots. The elements of the matrix $\boldsymbol{C}_V$ are the negative of the capacitive couplings between the plunger gates and the dots. In the notation of Supplementary Reference 1, the matrix $\boldsymbol{C} = \boldsymbol{C}_{cc}$ and $\boldsymbol{C}_V = \boldsymbol{C}_{cv}$. The effects of random background charges are accounted for by including a threshold chemical potential μ_{0i} on each dot or, equivalently, an offset voltage on each gate $V_{\mathrm{P}i}^{\mathrm{off}}$ (Supplementary Table 1). This offset is expressed in virtual gate voltage space as $u_i^{\mathrm{off}} \equiv \sum_j G_{ij} V_{\mathrm{P}j}^{\mathrm{off}}$.

The terms in the Hamiltonian can be extracted from measurements of pairwise charge stability diagrams for the array of dots (e.g. V_{P1} -vs- V_{P2}, V_{P2} -vs- V_{P3}, etc.). Diagonal elements of v_{ii} are determined by measuring the addition voltage ΔV_{21}^i of dot i and then using the simple relation

$$\frac{v_{ii}}{\alpha_1 |e|} = \frac{G_{ii}}{2}\Delta V_{21}^i. \tag{4}$$

ΔV_{21}^i can be directly extracted from charge stability diagrams (see Supplementary Figure 1). We have obtained an estimate of the lever arm for dot 1, $\alpha_1 = 0.12 \pm 0.01$, for the fully-configured array by using measurements of finite bias triangles with just dots 1 and 2 configured as a DQD.[1] This estimate is sufficient for our purposes because knowledge of the precise value of α_1 is not needed to

navigate the charge-stability space in virtual gate voltage space. The nearest-neighbor, off-diagonal terms of v_{ij}, offset voltages $V_{\mathrm{P}i}^{\mathrm{off}}$, diagonal and nearest-neighbor, off-diagonal elements of the matrix G_{ij} can be found by measuring the slopes of the charge transitions and triple point positions using the relations given in Supplementary Equations (6)–(10). Parameterizing charge transition ⓚ in Fig. 1 by the formula

$$V_{\mathrm{P}i} = r_k^i V_{\mathrm{P}i+1} + b_k^i, \tag{5}$$

we have the following relations

$$r_1^i = -\frac{G_{ii+1}}{G_{ii}},\ r_2^i = -\frac{G_{i+1i+1}}{G_{i+1i}},\ r_3^i = \frac{G_{i+1i+1} - G_{ii+1}}{G_{ii} - G_{i+1i}}, \tag{6}$$

$$\frac{v_{ii+1}}{\alpha_1|e|} = \frac{G_{ii}}{2}(b_5^i - b_1^i), \tag{7}$$

$$u_i^{\mathrm{off}} = G_{ii}b_1^i - \frac{v_{ii}}{\alpha_1|e|} + G_{ii-1}V_{\mathrm{P}i-1}, \tag{8}$$

$$u_{i+1}^{\mathrm{off}} = G_{i+1i}b_2^i - \frac{v_{i+1i+1}}{\alpha_1|e|} + G_{i+1i+2}V_{\mathrm{P}i+2}, \tag{9}$$

$$V_{\mathrm{P}i}^{\mathrm{off}} = \sum_j [\boldsymbol{G}^{-1}]_{ij}\, u_j^{\mathrm{off}}, \tag{10}$$

where u_i^{off} is the virtual gate voltage offset on dot i.

As an example of the data analysis, we measure the charge stability diagram associated with dots 1 and 2 as a function of the gate voltages $V_{\mathrm{P}1}$ and $V_{\mathrm{P}2}$, see Supplementary Figure 2. The dot 1 and dot 2 charge transitions are fit to extract the slopes r_1 and r_2. From the electron and hole triple point positions T_e and T_h, and these two slopes, we can compute r_3 and the intercepts b_{1-5}. From the extracted values we compute $\boldsymbol{G}$ and make the transformation to virtual gate voltage space.

When a new dot N is added to the array, we define new virtual gate voltages in terms of an expanded matrix $\boldsymbol{G}$ equal to the old $\boldsymbol{G}$ with the extensions $G_{NN} = 1$ and $G_{Ni} = G_{iN} = 0$ for $i \neq N$. We then tune the newly added dot into the single electron regime and take pairwise charge stability diagrams to determine the corrected matrix elements for $\boldsymbol{G}$ in the new device configuration. For the example of adding dot 4, shown in Supplementary Figure 3, we can see that the dot 1–dot 2 charge stability diagram in Supplementary Figure 3a is unaffected by the addition of the 4th dot. The dot 3 charge transition is slightly tilted in the dot 2–dot 3 charge stability diagram (see Supplementary Figure 3b), and the dot 3–dot 4 charge stability diagram appears as if it were acquired in plunger gate voltage space (see Supplementary Figure 3c). By fitting these data and adjusting $\boldsymbol{G}$, the charge transitions become orthogonal in each pairwise stability diagram when measured in virtual gate voltage space, as shown in Supplementary Figure 3d–f. Additional dots are added to the array following this procedure.

The final $\boldsymbol{v}$, $\boldsymbol{G}$, $\boldsymbol{C}$, and $\boldsymbol{C}_V$ matrices are given in Supplementary Figure 5. Since the cross-capacitances fall off rapidly in our device design these matrices only include terms up to the first off-diagonal elements. The V_{P} offsets for dot i, $V_{\mathrm{P}i}^{\mathrm{off}}$, and the initial charging energies for dot i, E_{ci}, are given in Supplementary Table 1. These charging energies are extracted from single dot stability diagrams taken as dots are added to the array and are similar to the charging energies previously reported on a similar device.[2] In a fully tuned configuration with barriers low enough for the shuttling experiment, the charging energies are expected to be lower as there is less confinement.

Using the relation $E_{ci} = 2v_{ii}$, we can verify this expected trend by comparing the initial values of E_{ci} from Supplementary Table 1 to our measurement of the matrix $\boldsymbol{v}$ for the fully tuned array shown in Supplementary Figure 5.

Once the capacitance matrices and voltage offsets are known it becomes possible to individually tune the chemical potential of each dot using voltage coordinates defined in virtual gate voltage space u_i through the relation

$$V_{\mathrm{P}i} = \sum_{ij} \left[\boldsymbol{G}^{-1}\right]_{ij} u_j. \tag{11}$$

In virtual gate voltage space, it is relatively straightforward to design pulse sequences that can shuttle electrons across the array. We use the approach described in the main text, where each interdot transition in the array is navigated in a pairwise fashion in three steps. In the first step, dots i and $i+1$ are brought from deep inside (1,0) close to the interdot charge transition. Here the indices (N_i, N_{i+1}) refer to the charge occupation of dots i and $i+1$. In step two, we adiabatically navigate the interdot charge transition to move the electron from dot i to dot $i+1$. In step three, we then move the system deep into the (0,1) charge configuration with the chemical potential of dot i brought above the Fermi energy of the source and drain electrodes. This prevents "back-flow" of electrons in the subsequent shuttling sequences in which an electron could hop from dot i to dot $i-1$ during the i to $i+1$ transfer sequence. The amplitudes of the pulses are computed from the addition energies to ensure that the lowest chemical potential of each dot during the dwell times are well away from the resonance condition for the addition of the next electron. This prevents accidental loading and shuttling of two electrons within the same dots.

In order to efficiently transfer an electron from one dot to the next during the shuttling sequence, the interdot charge transitions should be navigated at a rate slow compared to the interdot tunnel couplings. To more quantitatively determine the effect of non-adiabatic corrections on the shuttling efficiency we use an approach whereby we purposely introduce errors by lowering the interdot tunnel coupling, as shown in Supplementary Figure 4. If the tunneling rate is too slow compared to the speed of the pulse sequence,[3] then the electrons get stuck in the array more often and the current deviates from $I = ef$ at higher frequencies as is visible in the inset of Supplementary Figure 4. At our highest pulsing frequency 45.5 MHz for 4 dots, we start to detect errors in the electron shuttle when the tunnel coupling of one of the barriers in the device is lowered below 2.4 GHz. To avoid pumping errors, we set all tunnel couplings around 5 GHz. A Tektronix AWG5208 ran at 2 GS/s is used to generate the voltage pulses for the charge pumping sequence. Each AWG output is low-pass filtered using a Mini-circuits SBLP-117+ filter. The cryostat wiring is identical to that used in a previous experiment.[4]

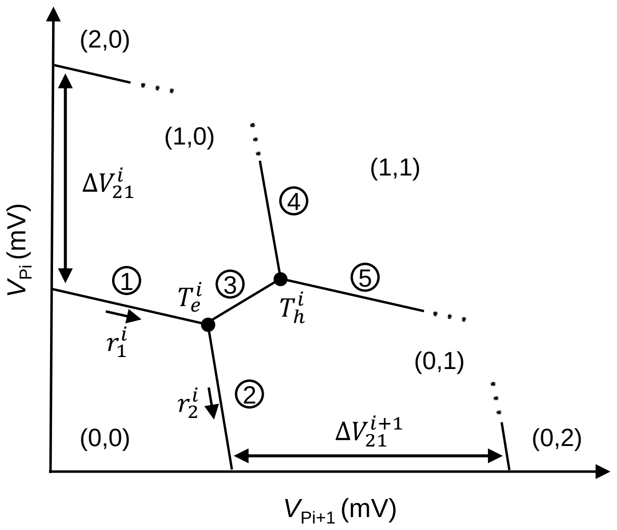

SUPPLEMENTARY FIG. 1. Double dot charge stability diagram of dots i and $i+1$. The charge transitions are defined by 5 lines, which are uniquely determined by the two slopes $r^i_{1,2}$ and the two triple point positions $T^i_{e,h}$. The charging energy of dot i can be extracted from the addition voltage ΔV^i_{21}.

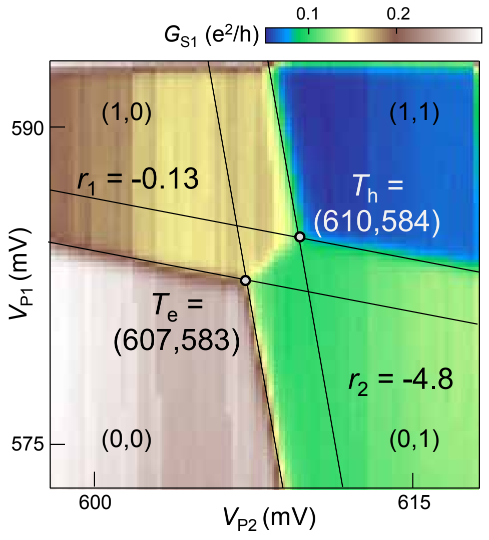

SUPPLEMENTARY FIG. 2. Double dot charge stability diagram with lines fit to the dot 1 and 2 charge transitions. The slopes of the dot 1 and 2 charge transitions, r_1 and r_2, and the triple point positions, T_e and T_h, are labeled. These parameters are used to calculate $\boldsymbol{G}$.

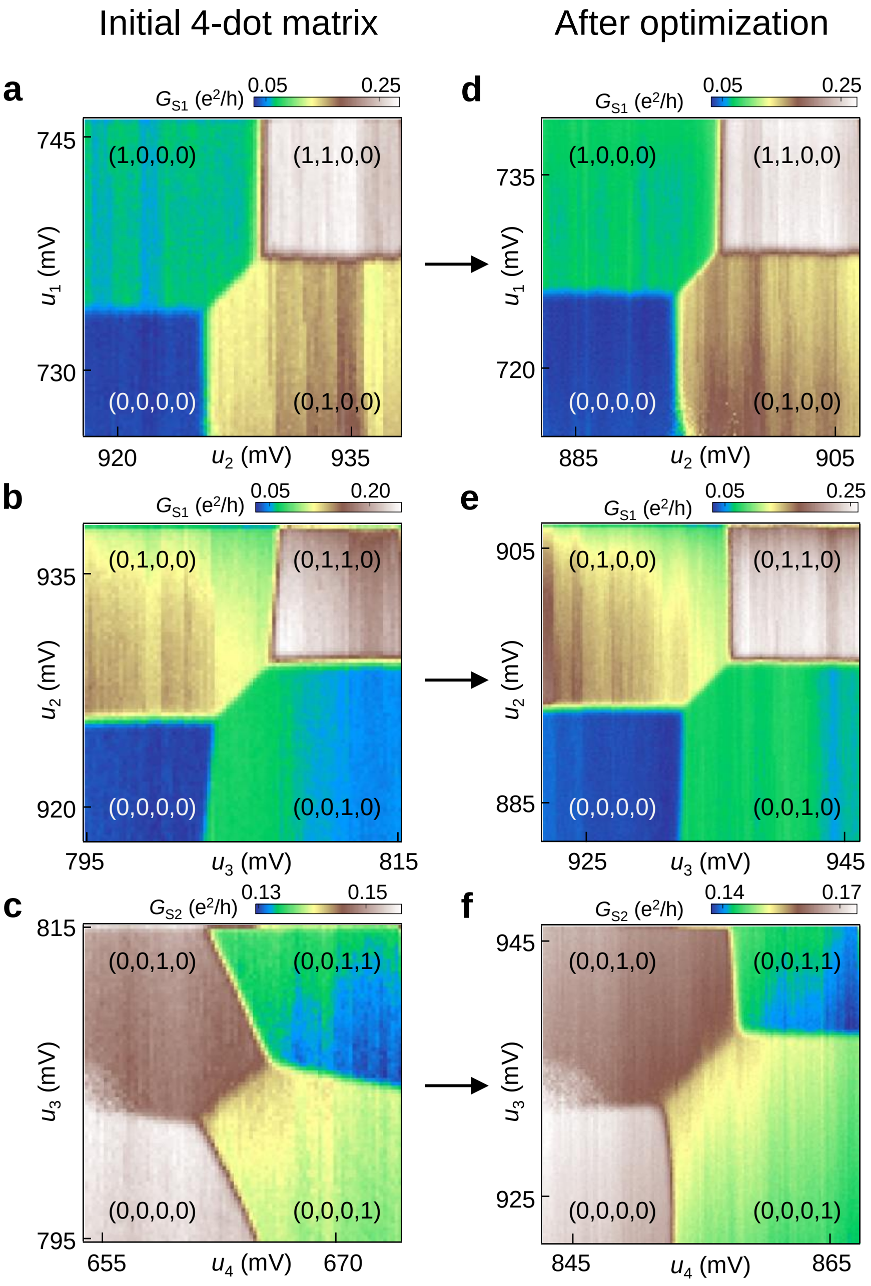

SUPPLEMENTARY FIG. 3. Pairwise charge stability diagrams measured on a 4-dot array. Panels (a–c) show the pairwise charge stability diagrams as measured before accounting for the cross-capacitance to plunger gate 4. Panels (d–f) show stability diagrams measured after $\boldsymbol{G}$ is recalibrated.

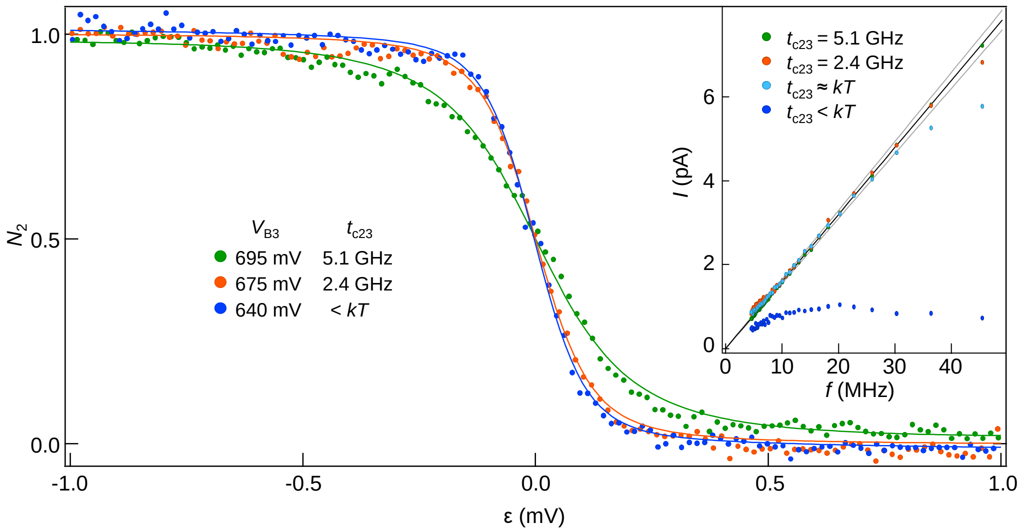

SUPPLEMENTARY FIG. 4. The number of electrons on dot 2, N_2, as a function of detuning, ϵ, for several values of the barrier gate voltage V_{B3}. The inset shows the pumped current I through 4 dots vs f, where f is the frequency of a full shuttling cycle, measured for different values of the interdot tunnel coupling t_{c23}. The solid black line shows the expected current $I = ef$, while the light grey lines above and below indicate the 3% gain error of our preamp. An error of about 6% is measured when shuttling at 45.5 MHz with $t_{c23} = 2.4$ GHz and more significant errors are detected when $t_{c23} < 2.4$ GHz. In these experiments, $T \approx 120$ mK.

$$v = \begin{bmatrix} 1.7 & 0.2 & - & - & - & - & - & - & - \\ 0.2 & 1.5 & 0.1 & - & - & - & - & - & - \\ - & 0.1 & 2.0 & 0.2 & - & - & - & - & - \\ - & - & 0.2 & 1.4 & 0.1 & - & - & - & - \\ - & - & - & 0.1 & 1.6 & 0.2 & - & - & - \\ - & - & - & - & 0.2 & 3.4 & 0.2 & - & - \\ - & - & - & - & - & 0.2 & 2.6 & 0.0 & - \\ - & - & - & - & - & - & 0.0 & 1.9 & 0.5 \\ - & - & - & - & - & - & - & 0.5 & 3.3 \end{bmatrix} \quad C = \begin{bmatrix} 49.1 & -5.4 & - & - & - & - & - & - & - \\ -5.4 & 54.7 & -3.1 & - & - & - & - & - & - \\ - & -3.1 & 41.1 & -6.1 & - & - & - & - & - \\ - & - & -6.1 & 56.8 & -2.2 & - & - & - & - \\ - & - & - & -2.2 & 50.0 & -2.3 & - & - & - \\ - & - & - & - & -2.3 & 24.1 & -2.3 & - & - \\ - & - & - & - & - & -2.3 & 31.1 & -0.8 & - \\ - & - & - & - & - & - & -0.8 & 43.2 & -6.2 \\ - & - & - & - & - & - & - & -6.2 & 25.3 \end{bmatrix}$$

$$G = \begin{bmatrix} 1 & 0.21 & - & - & - & - & - & - & - \\ 0.19 & 1.09 & 0.15 & - & - & - & - & - & - \\ - & 0.28 & 1.13 & 0.10 & - & - & - & - & - \\ - & - & 0.32 & 0.77 & 0.05 & - & - & - & - \\ - & - & - & 0.20 & 0.93 & 0.12 & - & - & - \\ - & - & - & - & 0.37 & 1.39 & 0.19 & - & - \\ - & - & - & - & - & 0.42 & 1.37 & 0.12 & - \\ - & - & - & - & - & - & 0.17 & 1.13 & 0.40 \\ - & - & - & - & - & - & - & 0.27 & 1.23 \end{bmatrix} \quad C_V = \begin{bmatrix} -5.8 & -0.6 & - & - & - & - & - & - & - \\ -0.6 & -6.9 & -0.6 & - & - & - & - & - & - \\ - & -0.9 & -5.3 & 0.1 & - & - & - & - & - \\ - & - & -1.3 & -5.1 & -0.1 & - & - & - & - \\ - & - & - & -1.0 & -5.5 & -0.3 & - & - & - \\ - & - & - & - & -0.8 & -3.9 & -0.2 & - & - \\ - & - & - & - & - & -1.2 & -5.1 & -0.4 & - \\ - & - & - & - & - & - & -0.7 & -5.7 & -1.1 \\ - & - & - & - & - & - & - & 0.0 & -3.4 \end{bmatrix}$$

SUPPLEMENTARY FIG. 5. Measurements of the interaction matrix $\boldsymbol{v}$ (units of meV), the dimensionless matrix $\boldsymbol{G}$, and the capacitance matrices $\boldsymbol{C}$ and $\boldsymbol{C}_V$ (units of aF) using iterative correction approach.

	$V_{\mathrm{P}i}^{\mathrm{off}}$ (mV)	E_{ci} (meV)
Dot 1	583	5.4 ± 0.3
Dot 2	605	5.6 ± 0.3
Dot 3	588	5.1 ± 0.5
Dot 4	660	4.6 ± 0.5
Dot 5	707	5.3 ± 0.6
Dot 6	750	5.2 ± 0.2
Dot 7	650	4.9 ± 0.5
Dot 8	640	5.1 ± 0.3
Dot 9	635	5.3 ± 0.4

SUPPLEMENTARY TABLE 1: The plunger offset voltages $V_{\mathrm{P}i}^{\mathrm{off}}$ and charging energies E_{ci} are listed for the 9 dots in the array. The charging energies are extracted from single dot charge stability diagrams that are acquired as dots are added to the array. In this configuration, we use an estimated lever arm of $\alpha_1 = 0.12$ for each dot.

[1] W. G. van der Wiel, S. De Franceschi, J. M. Elzerman, T. Fujisawa, S. Tarucha, and L. P. Kouwenhoven, *Electron transport through double quantum dots*, Rev. Mod. Phys **75**, 1 (2003).

[2] D. M. Zajac, T. M. Hazard, X. Mi, E. Nielsen, and J. R. Petta, *Scalable gate architecture for a one-dimensional array of semiconductor spin qubits*, Phys. Rev. Appl. **6**, 054013 (2016).

[3] L. J. Geerligs, S. M. Verbrugh, P. Hadley, J. E. Mooij, H. Pothier, P. Lafarge, C. Urbina, D. Esteve, and M. H. Devoret, *Single Cooper pair pump*, Z. Phy. B **85**, 349 (1991).

[4] D. M. Zajac, A. J. Sigillito, M. Russ, F. Borjans, J. M. Taylor, G. Burkard, and J. R. Petta, *Resonantly driven CNOT gate for electron spins*, Science **359**, 439 (2018).
